# Does functional homogenization accompany taxonomic homogenization of British birds and how do biotic factors and climate affect these processes?

**DOI:** 10.1002/ece3.4267

**Published:** 2018-06-27

**Authors:** Hannah J. White, W. Ian Montgomery, Lenka Storchová, David Hořák, Jack J. Lennon

**Affiliations:** ^1^ School of Biological Sciences Queen's University Belfast Belfast UK; ^2^ School of Biology and Environmental Science Earth Institute University College Dublin Dublin Ireland; ^3^ Institute of Global Food Security (IGFS) Queen's University Belfast Belfast UK; ^4^ Department of Ecology Charles University Prague Czech Republic

**Keywords:** biotic homogenization, climate, ecological traits, functional diversity, local species distribution

## Abstract

Environmental change has reshuffled communities often causing taxonomic homogenization rather than differentiation. Some studies suggest that this increasing similarity of species composition between communities is accompanied by an increase in similarity of trait composition—functional homogenization—although different methodologies have failed to come to any consistent conclusions. Functional homogenization could have a large effect on ecosystem functioning and stability. Here, we use the general definition of homogenization as “reduced spatial turnover over time” to compare changes in Simpson's beta diversity (taxonomic turnover) with changes in Rao's quadratic entropy beta diversity (functional turnover) in British breeding birds at three spatial scales. Using biotic and climatic variables, we identify which factors may predispose a site to homogenization. The change in turnover measures between two time periods, 20 years apart, was calculated. A null model approach was taken to identify occurrences of functional homogenization and differentiation independent of changes in taxonomic turnover. We used conditional autoregressive models fitted using integrated nested Laplace approximations to determine how environmental drivers and factors relating to species distributions affect changes in spatial turnover of species and functional diversity. The measurement of functional homogenization affects the chance of rejection of the null models, with many sites showing taxonomic homogenization unaccompanied by functional homogenization, although occurrence varies with spatial scale. At the smallest scale, while temperature‐related variables drive changes in taxonomic turnover, changes in functional turnover are associated with variation in growing degree days; however, changes in functional turnover become more difficult to predict at larger spatial scales. Our results highlight the multifactorial processes underlying taxonomic and functional homogenization and that redundancy in species traits may allow ecosystem functioning to be maintained in some areas despite changes in species composition.

## 1 INTRODUCTION

The effects of environmental change on ecological communities include an increase in compositional similarity between many areas, a process known as biotic homogenization (McKinney & Lockwood, 1999; Olden & Rooney, 2006). Biotic homogenization is projected to have both ecological and evolutionary consequences, including an effect on ecosystem resilience to environmental perturbations (Olden, Poff, Douglas, Douglas & Fausch, 2004). The degree of homogenization varies across space (Olden, Poff & McKinney, 2006), and certain areas may be particularly susceptible due to a combination of biotic and abiotic factors (Olden & Poff, 2003; Ross, Woodin, Hester, Thompson & Birks, 2012).

Taxonomic homogenization, one form of biotic homogenization, can be identified by comparing spatial turnover of species between time periods; a decrease in spatial turnover indicates homogenization, whereas an increase indicates differentiation (Baiser & Lockwood, 2011; McKinney & Lockwood, 1999; Olden et al., 2004; Tobias & Monika, 2011). However, homogenization can also comprise increasing similarity of community trait composition: a process known as “functional homogenization” (Tobias & Monika, 2011). Traits are an important component of biodiversity due to their role in driving ecosystem stability and functioning (Díaz & Cabido, 2001; Olden et al., 2004), shaping species distributions (Pollock, Morris & Vesk, 2012), and determining responses to environmental change (Flynn et al., 2009; Newbold et al., 2012). Understanding the turnover of traits in space and time, therefore, has been recognized as an essential area of investigation to determine whether changes in taxonomic turnover are accompanied by changes in functional turnover, or whether functional redundancy may ensure ecosystem functioning is maintained despite losses in taxonomic diversity (Villéger, Grenouillet & Brosse, 2014). Previous studies failed to find a consistent relationship between functional and taxonomic homogenization with results varying between location, environmental pressures, and focal taxa (Abadie, Machon, Muratet & Porcher, 2011; Devictor, Julliard, Couvet, Lee & Jiguet, 2007; Monnet et al., 2014; Reif et al., 2013; Sonnier, Johnson, Amatangelo, Rogers & Waller, 2014). Many of these only use a proxy for functional homogenization, that is, mean community specialization. This method assumes that generalist species colonize an area and outcompete specialist species, thus decreasing the mean specialization of the community (Clavel, Julliard & Devictor, 2011; Davey, Chamberlain, Newson, Noble & Johnston, 2012). Using mean specialization, however, ignores similarity between communities (Gosselin, 2012), which is integral to the general definition of homogenization as an increase in spatial similarity of genetic, functional, or taxonomic diversity in time (Olden & Rooney, 2006; Olden et al., 2004). More recently, a handful of studies have measured functional homogenization by calculating the difference in functional (dis)similarity between communities over time (Baiser & Lockwood, 2011; Monnet et al., 2014; Sonnier et al., 2014). Here, we use a similar “difference in turnover” method to incorporate a variety of ecological traits into a measure comparable with that of taxonomic homogenization.

Climate modifies the local environment, leading to both taxonomic and functional homogenization (Meynard et al., 2011; Sonnier et al., 2014). For example, areas which have undergone a long‐term increase in minimum temperature are expected to exhibit homogenization as species adapted to warmer conditions dominate the landscape by shifting their range (Devictor, Julliard, Couvet & Jiguet, 2008; Powney, Cham, Smallshire & Isaac, 2015). Additionally, temperature, precipitation, and soil acidity have all been identified as drivers of taxonomic homogenization in plants (Ross et al., 2012), indicating the importance of environmental factors to changes in community similarity. Much of the literature concerning environmental change and biodiversity focuses on mean measures (e.g., Moreno‐Rueda & Pizarro, 2008); however, with climate change, a higher incidence of extreme events is expected with regards to temperature and precipitation (Jentsch & Beierkuhnlein, 2008). It is, therefore, important to consider more criteria than just mean values of climatic parameters to forecast effects of climate change on biodiversity (Buckley & Kingsolver, 2012).

Biotic factors may also affect homogenization. Conceptual models suggest that initial community similarity, species richness, and ratio of invading species to those that undergo local extinction may influence changes in spatial turnover of species over time (Olden & Poff, 2003). The effect of biotic factors on homogenization using empirical data, however, remains to be investigated.

Here, we use data on British bird distributions from two atlas datasets collected 20 years apart to map changes in neighborhood turnover of species and traits using a moving window (similar to Barnagaud et al., 2017; McKnight et al., 2007) and address two main objectives: (a) determine whether functional homogenization accompanies taxonomic homogenization, improving on previous methods by measuring how spatial turnover of taxonomic and functional diversity changes between the two time periods, and (b) identify climatic and biotic factors that influence the vulnerability of communities to homogenization. As the drivers of avian *β* diversity differ between functional and taxonomic diversity (Meynard et al., 2011), it is likely that the drivers of change in *β* diversity, or turnover, over time also vary between functional and taxonomic diversity. To address this, we use a range of climatic variables covering multiple physical aspects of the environment along with three biotic factors: mean binomial variance (relating to local species occurrence), species richness in the earlier atlas, and functional diversity in the earlier atlas. The mean binomial variance of species’ local ranges is likely to affect the ability of a species to increase its local range, that is, if the majority of species are present in 50% of the neighboring squares, then it is more likely that sufficient numbers of species will be able to increase or decrease their local range size so that their occurrences are more homogeneous and, therefore, contribute to taxonomic homogenization. On the other hand, if the majority of species in the neighboring area are either locally common or locally rare, then it is less likely that there will be a negative change in *β* diversity as the area is already taxonomically similar. We expect, therefore, that areas are more susceptible to taxonomic homogenization if the central tendency of species’ local ranges is intermediate, that is, the mean binomial variance of all the species in the area is high.

Given the above, we test the hypotheses: (a) functional and taxonomic homogenization occur independently of each other; and (b) biotic factors have a larger effect on taxonomic homogenization due to the geographical limits on species dispersal potential, while climatic variables have a larger effect on functional homogenization due to trait–environment associations (Cormont, Vos, Van Turnhout, Foppen & ter Braak, 2011). Identifying key promoters of homogenization will help inform policymakers to prioritize areas which are vulnerable to future homogenization for conservation planning and, therefore, help mitigate the adverse consequences of climate change (Rooney, Olden, Leach & Rogers, 2007).

## 2 MATERIALS AND METHODS

### 2.1 Data

British bird distribution data at the 10 × 10 km (hectad) scale for the periods 1968–1972 and 1988–1991 were obtained from two atlases of breeding birds (Gibbons, Reid & Chapman, 1993; Sharrock, 1976; respectively). We excluded marine species and rare vagrants from the analyses. Squares with less than 50% land or no immediately neighboring squares were also excluded leaving a total of 167 species recorded in 2,253 sites across Great Britain. Throughout the analyses, the focal square is defined as each hectad in turn and neighboring squares as each immediately surrounding square, that is, one focal square and the eight neighboring squares would form a 30 × 30 km grid. This moving window approach measures the neighborhood turnover of each individual hectad and matches the methods used by Barnagaud et al. (2017), but at a finer resolution. We also considered multiple focal square sizes by aggregating the hectad data, increasing the scale of the analyses to a focal square of 30 × 30 km and 90 × 90 km. Often, differences in recorder effort can confound analyses of citizen science data; however, recorder effort for the two atlases used here is considered intensive and relatively consistent (Evans, Greenwood & Gaston, 2005).

Trait data was obtained from the European Bird Trait Database (Storchová, Hořák & Hurlbert, 2018). Body mass, clutch size, age at first breeding, young developmental type (altricial, semialtricial, or precocial), nesting behavior (solitary nester, semicolonial, and/or colonial), nest type (ground, hole, open arboreal, closed arboreal, ground closed, and/or nest parasite), migratory behavior, and diet were selected for our analyses as they “impact fitness indirectly via effects on growth, reproduction, and survival” (Violle et al., 2007), have complete coverage of the species included in the study, are important response or effect traits (Petchey & Gaston, 2006), and minimize redundant information between traits (Lefcheck, Bastazini & Griffin, 2014). Although they are technically behavioral characteristics, migratory status and diet were included as traits, as in other studies of vertebrate functional diversity (Luck, Carter & Smallbone, 2013).

### 2.2 Variable calculation

Three biotic variables were included in each analysis; taxonomic and functional diversity in the focal square in the earlier of the two atlases, and mean binomial variance of local species distributions, details of which follow. Species richness was included as a measure of taxonomic diversity in the earlier atlas. Functional diversity was calculated using Rao's quadratic entropy (Rao Q; Botta‐Dukát, 2005). Among the large number of functional diversity measures currently used within the literature (Mouchet et al., 2005), Rao Q was chosen as it can be used to calculate both *α* and *β* components of diversity. Rao Q measures the functional distance between two randomly selected individuals within a community (Ricotta, 2005). We used Gower distances to measure functional distances which can incorporate both continuous and discrete traits. Rao Q was calculated using the *dbFD* function in the R package FD (Laliberté & Legendre, 2010; Laliberté, Legendre & Shipley, 2014).

The mean binomial variance represents the local commonness or rarity of species locally. This measure was chosen as it reflects the distribution of species’ local occurrences which we might expect to influence species turnover. Mean binomial variance is calculated by taking the average of the binomial variance (*npq*—where *n* = number of hectads, *p* = number surrounding hectads in which the species is present, and *q* = number of surrounding hectads in which the species is absent) of occurrence of all species found in the focal square and its neighboring squares (i.e., within a grid of 30 × 30 km). This measure was then recalculated with a focal square of 30 × 30 km and neighborhood of 90 × 90 km, and again with a focal square of 90 × 90 km and neighborhood of 270 × 270 km. This measure has a maximum value if all species occurring locally are present in 50% of the neighboring squares and a minimum value if either all species in the focal square are locally ubiquitous or locally absent.

Monthly temperature and precipitation data were downloaded from UKCP09 (metoffice.gov.uk/climatechange/science/monitoring/ukcp09) for the years 1961–1990 and used to calculate climatic variables relating to:

1. Minimum temperature—the mean daily minimum temperature for the coldest annual month;
2. Drought—potential evapotranspiration minus the total annual precipitation using the method of Burt and Shahgedanova (1998); and
3. Growing degree days—the mean total accumulated temperature above a threshold of 5.5°C.

For each of these attributes the following measures were calculated:

1. Mean—average of the 30 (each year) values;
2. Variance—residual variance of an observed least squares regression through the data;
3. Lag autocorrelation—correlation between each variable at time *t* and time *t* − 1;
4. Long‐term trend—slope of observed least squares regression line fitted through the time series;
5. Fat tail—relative frequency of more extreme climatic events, that is, time spent in the tail of the distribution of values relative to the time spent in the central distribution mass calculated as (*Q* 0.975 − *Q* 0.025)/(*Q* 0.875 − *Q* 0.125), where *Q* is the quantile function (Brys, Hubert & Struyf, 2006);

In total, this produces 15 climatic variables included within the analyses.

### 2.3 Turnover calculations

We measured taxonomic turnover using the modified Simpson's index, *β*_sim_ (Lennon, Koleff, Greenwood & Gaston, 2001):

$$\beta_{\mathrm{sim}}=\frac{\min(b,c)}{\min(b,c)+a}$$

where *a* is the number of species found in both the focal community and the neighboring community, *b* is the number of species found only in the neighboring community and not the focal community, and *c* is the number of species found in the focal community but absent from the neighboring community. *β*_sim_ was found to perform best in a review of 24 *β* diversity measures (Koleff, Gaston & Lennon, 2003). It is a “narrow” sense measure of *β* diversity which focuses on compositional differences in communities independent of any species richness gradients (Lennon et al., 2001) and satisfies the requirements of symmetry, homogeneity, and nestedness of robust *β* diversity measures (Koleff et al., 2003).

Functional turnover was calculated using the decomposition of Rao Q, which we call *β*_rao_. This measures the gain in functional diversity when communities are pooled, that is, the difference between the dissimilarity of two random individuals in the whole region (the pooled communities) and the dissimilarity of two individuals within communities (De Bello et al., 2009). We used the R function *betaQmult* provided in Villéger, Ramos Miranda, Flores Hernandez and Mouillot (2012) to calculate *β*_rao_. *β*_rao_ was calculated for the focal square and each of its neighbors (maximum 8) in turn, and the mean of each taken. This accounted for fewer neighbors for coastal squares. We applied a moving window algorithm so that each square within the dataset was included as the focal square in the calculations of turnover for both atlases. Two additional measures of functional turnover, nearest functional neighbor and mean functional dissimilarity (Sonnier et al., 2014; Swenson et al., 2012) between neighboring hectads were also calculated to test whether the occurrence of functional homogenization differs with the measure of functional turnover used. These measures use functional distances between species, similar to *β*_rao_, to calculate functional dissimilarity between communities; however, their construction and interpretation are less similar to *β*_sim_. The details and results of nearest functional neighbor and mean functional dissimilarity measures of functional turnover are provided in the supplementary information.

### 2.4 Analyses

Two sets of analyses were carried out; investigating the occurrence of homogenization and investigating the drivers of homogenization. For both sets of analyses, the degree of homogenization for all turnover measures was calculated as the spatial turnover in the second atlas minus the spatial turnover in the first atlas. As *β*_sim_ and *β*_rao_ are dissimilarity measures, negative differences represent homogenization, while positive values indicate differentiation between the two time periods. All analyses were carried out at the three spatial scales: 10 × 10; 30 × 30; and 90 × 90 km.

We took a null model approach to test the presence of functional homogenization irrespective of taxonomic homogenization. We used the random assembly model, which is a trait‐level null model (Morlon et al., 2010) where species row names are shuffled in the species‐by‐trait matrix using the independent swap algorithm (Gotelli & Entsminger, 2003) to create 999 new random species‐by‐trait matrices. This constrained randomization approach maintains species richness, species turnover, spatial structure of species distributions, trait ranges, and trait covariances (Swenson et al., 2012). The random trait matrices were used to calculate *β*_rao_ for both atlases from which change between the two atlases was calculated. This gave us a null distribution of changes in functional turnover to address the question of whether functional homogenization accompanies taxonomic homogenization. We calculated *p* values for the location of the observed change in turnover in the null probability distribution to determine whether to reject the null hypothesis using a two‐tailed test.

We used intrinsic conditional autoregressive models, which account for spatial autocorrelation within the error term, under Bayesian inference using the Integrated Nested Laplace Approximation (INLA) to model changes in functional turnover (*β*_rao_) as a function of change in taxonomic turnover (*β*_sim_) using a normally distributed, uninformative prior with a precision of 0.001. Spatial errors were given log‐gamma priors with a precision of 0.005. Plotting these relationships at each scale allowed the identification of homogenization and differentiation processes within each square.

To identify specific drivers of homogenization we regressed climatic and biotic variables against the changes in turnover calculated above. These were combined into a multimodel information theoretic approach (Burnham & Anderson, 2002) using a restricted set of three models, shown in Table 1, and compared using Deviance Information Criterion (DIC) which provides a measure of model fit. Although this measure can underpenalize models with a complex random error structure, we chose this criterion over Watanabe‐Akaike information criterion (WAIC) which assumes independent observations and, therefore, is not appropriate for our spatially structured data (Hooten & Hobbs, 2014). This combination of models was selected to test the hypothesis that biotic variables drive changes in taxonomic turnover, while climatic variables drive changes in functional turnover. Out of the vast number of variable combinations possible, the restricted set of models included in this analysis do not favor the selection of one set of variables over the other. Due to the spatial nature of the data, we again used intrinsic conditional autoregressive models which account for the spatial structure within the residuals to model the change in spatial turnover between the two time periods as a function of the climatic and biotic variables outlined above. We took a Bayesian inference approach to these models using INLA. Latitude and longitude were also included as fixed effects in all models; this relaxes the assumption of intrinsic conditional autoregressive models that spatial errors are stationary (Beale, Brewer & Lennon, 2014; Beale, Lennon, Yearsley, Brewer & Elston, 2010). This improves the accuracy of credible interval estimation (Beale et al., 2014). All variables were scaled and centered to aid with convergence and to enable comparisons of effect sizes post hoc. All models were scaled to have a generalized variance equal to 1, which reduced the number of effective parameters, and were fitted with a Gaussian likelihood. Covariates were given normally distributed, uninformative priors with a precision of 0.001, while spatial errors were fitted with log‐gamma priors and a precision of 0.005. Analyses were carried out in R v3.2.2 using the package R‐INLA (Rue, Martino & Chopin, 2009).

**Table 1:** Table of variables included in each of the models for multimodel inference

	Lat	Long	SR	Rao Q	Mean bin. var.	Min. temp.	Drought	GDD	varT	varD	varG	autoT	autoD	autoG	LTTT	LTTD	LTTG	FtailT	FtailD	FtailG
Full model	✓	✓	✓	✓	✓	✓	✓	✓	✓	✓	✓	✓	✓	✓	✓	✓	✓	✓	✓	✓
Biotic model	✓	✓	✓	✓	✓															
Climate model	✓	✓				✓	✓	✓	✓	✓	✓	✓	✓	✓	✓	✓	✓	✓	✓	✓

*Note*

auto*x*: lag autocorrelation; *D*: variables relating to drought; Drought: mean drought; Ftail*x*: fat tail measure of extreme events; *G*: variables relating to growing degree days; GDD: mean growing degree days; Lat: latitude; Long: longitude; LTT*x*: detrended long‐term trend; Mean bin. var.: mean binomial variance; Min. temp.: mean minimum temperature; Rao Q: Rao's quadratic entropy; SR: species richness; *T*: variables relating to minimum temperature; var*x*: variance.

## 3 RESULTS

### 3.1 Occurrence of functional homogenization

**Figure 1:**
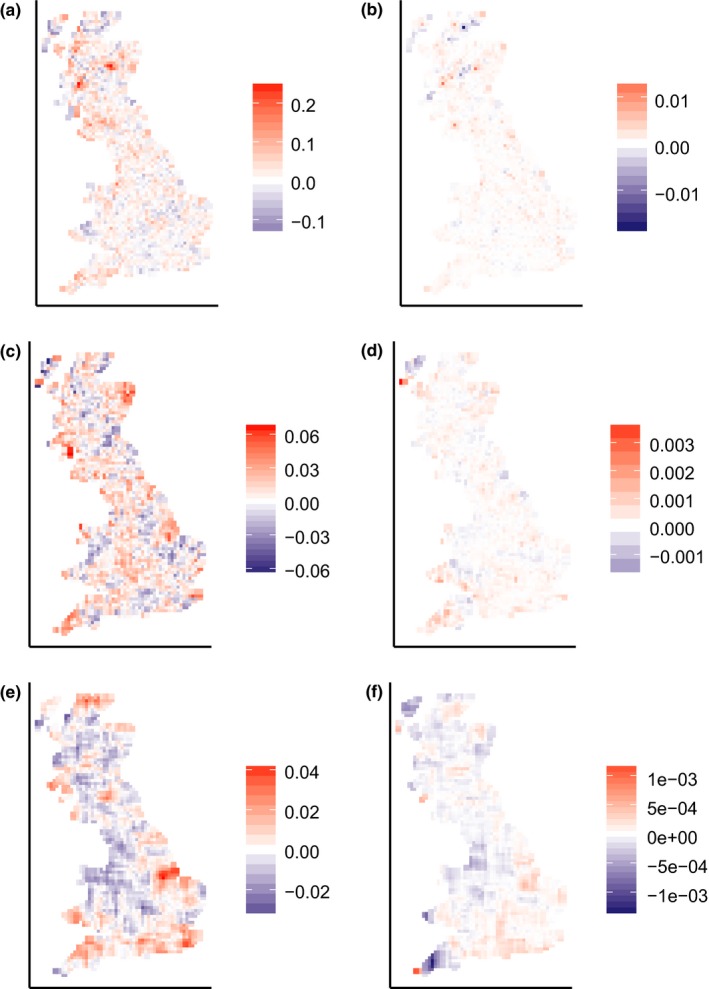
Maps showing change in *β*_sim_ (a,c,e) and *β*_rao_ (b,d,f) of British avifauna in squares of 10 × 10 km (top row), 30 × 30 km (middle row), and 90 × 90 km (bottom row) between two time periods 20 years apart

**Figure 2:**
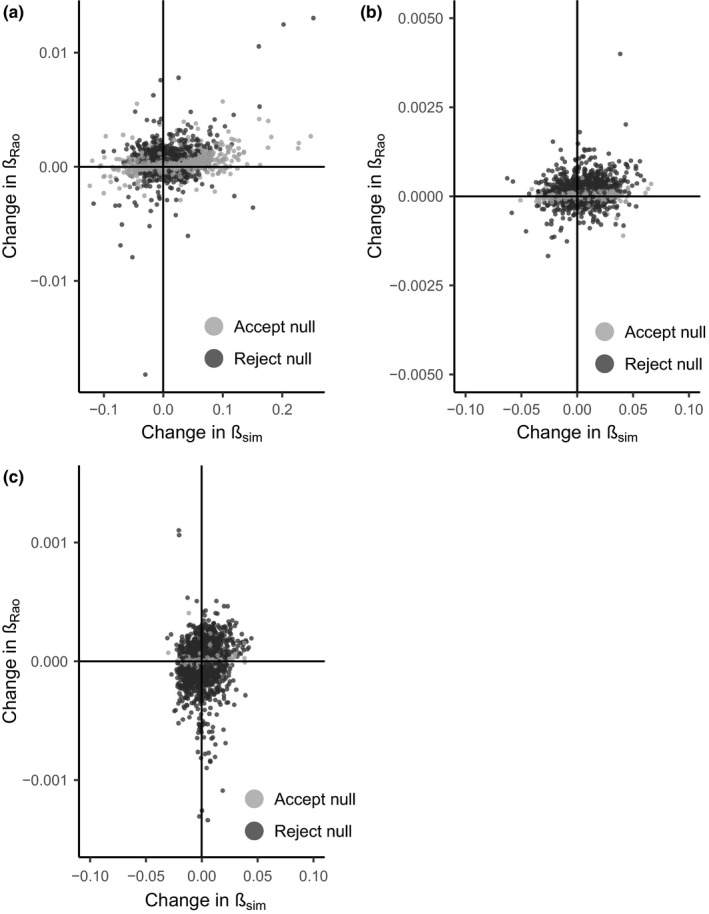
Scatter plot of change in functional turnover (*β*_rao_) plotted against change in taxonomic turnover (*β*_sim_) at three spatial scales (a) 10 km, (b) 30 km, and (c) 90 km squares. Dark points indicate sites where the change in functional turnover is different from that expected from the null distribution generated, and therefore, we reject the null model. The null distribution was generated using a random name swap algorithm to randomize the species‐by‐trait matrix for calculation of *β*_rao_

Changes in *β*_sim_ and *β*_rao_ varied in space (Figure 1). Change in *β*_rao_ varied with change in *β*_sim_ at the hectad scale [median = 0.0083, credible intervals (CI) = (0.0038, 0.013); Figure 2a] but not at either of the larger scales investigated [30 km scale: median = 0.0055, CI = (−0.0046, 0.0155); 90 km scale: median = 0.00190, CI = (−0.014, 0.0178); Figure 2b,c]. The null model of random change in *β*_rao_ was rejected in 25.6% of squares at the hectad scale, 54.8% of squares at the 30 km scale, and 81.5% sites at the 90 km scale, meaning that as spatial scale increases, functional turnover changes are more than expected by chance given changes in taxonomic turnover. The observed occurrences of homogenization and differentiation of species and traits varied with spatial scale (Tables 2, 3, 4). At the hectad scale, where the change in *β*_rao_ differed from that expected from the null model, the majority of squares (438 of 599) showed an increase in functional turnover between the two time periods, indicating functional differentiation. Most of these sites (349 of 599) also exhibited taxonomic differentiation through an increase in *β*_sim_; however, 189 sites showed functional differentiation but taxonomic homogenization, that is, an increase in *β*_rao_ but a decrease in *β*_sim_. The largest spatial scale (90 × 90 km) was the only scale at which taxonomic homogenization was more frequently associated with functional homogenization rather functional differentiation. The use of additional measures of functional turnover, that is, nearest functional neighbor and mean functional dissimilarity, showed that the occurrence of functional homogenization depends on the measure of functional turnover used (Supporting Information Appendix S2).

**Table 2:** Table of counts of squares where changes in *β*_rao_ differed from expected given a null distribution of changes at the 10 × 10 km scale

	Functional homogenization	Functional differentiation
Taxonomic homogenization	61	189
Taxonomic differentiation	100	249

**Table 3:** Table of counts of squares where changes in *β*_rao_ differed from expected given a null distribution of changes at the 30 × 30 km scale

	Functional homogenization	Functional differentiation
Taxonomic homogenization	152	305
Taxonomic differentiation	216	567

**Table 4:** Table of counts of squares where changes in *β*_rao_ differed from expected given a null distribution of changes at the 90 × 90 km scale

	Functional homogenization	Functional differentiation
Taxonomic homogenization	618	321
Taxonomic differentiation	433	463

### 3.2 Drivers of changes in turnover

**Figure 3:**
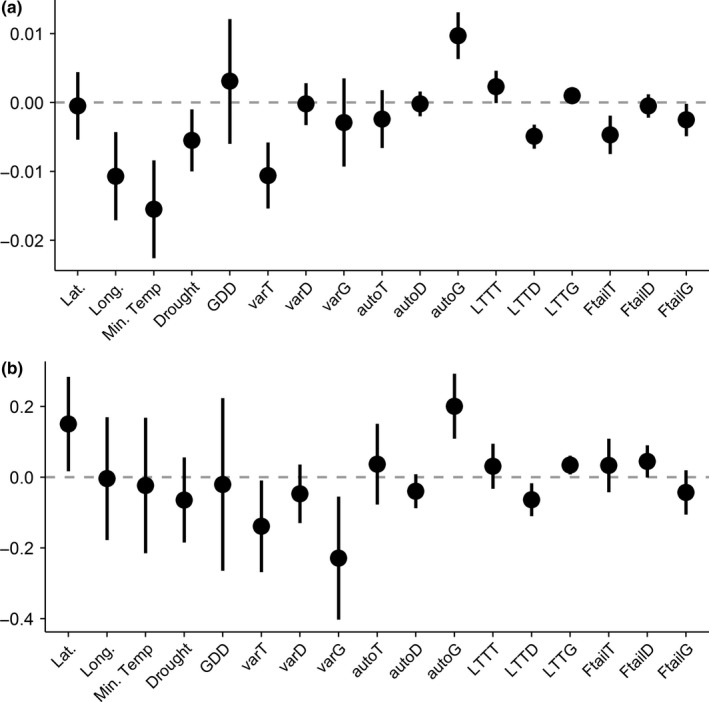
Coefficient plots of the climate only model for the analyses carried out at the 10 × 10 km scale, which was the best‐fitting model determined using DIC, for (a) change in *β*_sim_ and (b) change in *β*_rao_. Points indicate the median estimate of the posterior distribution, and lines represent the 95% credible interval. Where the credible intervals cross the dashed 0 line that particular independent variable does not have a substantial effect on the dependent variable. auto*x*: lag autocorrelation; *D*: variables relating to drought; Drought: mean drought; Ftail*x*: fat tail measure of extreme events; *G*: variables relating to growing degree days; GDD: mean growing degree days; Lat.: latitude; Long.: longitude; LTT *x*: detrended long‐term trend; Min. temp.: mean minimum temperature; *T*: variables relating to minimum temperature; var*x*: variance

**Figure 4:**
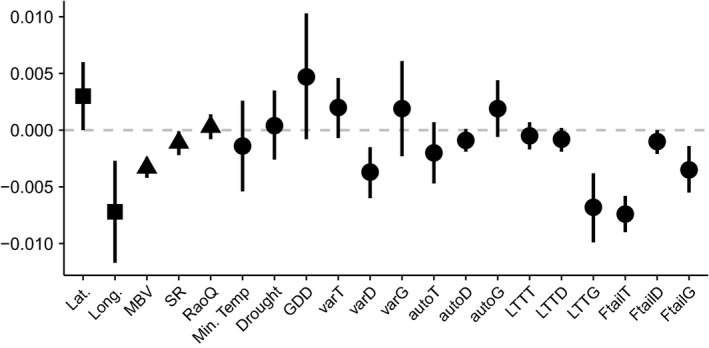
Coefficient plot of the full model for change in *β*_sim_ at the 30 × 30 km scale. This was the best‐fitting model, determined using DIC, at this scale. Points indicate the median estimate of the posterior distribution, and lines represent the 95% credible interval. Where the credible intervals cross the dashed 0 line that particular independent variable does not have a substantial effect on the dependent variable. Squares denote spatial covariates, triangles denote biotic covariates, and circles denote climate covariates. auto*x*: lag autocorrelation; *D*: variables relating to drought; Drought: mean drought; Ftail*x*: fat tail measure of extreme events; *G*: variables relating to growing degree days; GDD: mean growing degree days; Lat.: latitude; Long.: longitude; LTT *x*: detrended long‐term trend; MBV: mean binomial variance; Min. temp.: mean minimum temperature; RaoQ: Rao's quadratic entropy; SR: species richness; *T*: variables relating to minimum temperature; var*x*: variance

**Figure 5:**
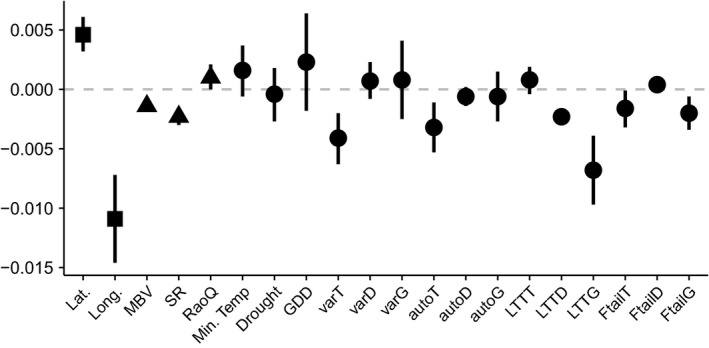
Coefficient plot of the full model for change in *β*_sim_ at the 90 × 90 km scale. This was the best‐fitting model, determined using DIC, at this scale. Points indicate the median estimate of the posterior distribution, and lines represent the 95% credible interval. Where the credible intervals cross the dashed 0 line that particular independent variable does not have a substantial effect on the dependent variable. Squares denote spatial covariates, triangles denote biotic covariates and circles denote climate covariates. auto*x*: lag autocorrelation; *D*: variables relating to drought; Drought: mean drought; Ftail*x*: fat tail measure of extreme events; *G*: variables relating to growing degree days; GDD: mean growing degree days; Lat.: latitude; Long.: longitude; LTT *x*: detrended long‐term trend; MBV: mean binomial variance; Min. temp.: mean minimum temperature; RaoQ: Rao's quadratic entropy; SR: species richness; *T*: variables relating to minimum temperature; var*x*: variance

The climate only model had the lowest DIC for both change in *β*_sim_ and change in *β*_rao_ at the hectad scale (Table 5), meaning that biotic variables were excluded from both best‐fitting models. For the two larger spatial scales, the full model performed best for changes in *β*_sim_, while the biotic only model performed best for changes in *β*_rao_. The covariates with the largest effect sizes varied between turnover measures and spatial scale (Figures 3, 4, 5).

**Table 5:** Table showing the models ranked by DIC for changes in each of the measures of turnover and the differences in DIC from the best performing model

Scale	Turnover measure	Model	DIC	ΔDIC
10 × 10 km	Change in *β*_sim_	Climate	−14779.87	0
		Full	−14658.46	121.41
		Biotic	−13430.89	1348.98
	Change in *β*_rao_	Climate	−11056.94	0
		Biotic	−11047.75	9.19
		Full	−11044.92	12.02
30 × 30 km	Change in *β*_sim_	Full	−17071.12	0
		Biotic	−17017.53	53.59
		Climate	−16828.00	243.12
	Change in *β*_rao_	Biotic	−23153.78	0
		Full	−23148.17	5.61
		Climate	−23142.58	11.2
90 × 90 km	Change in *β*_sim_	Full	−18487.43	0
		Climate	−18352.79	134.64
		Biotic	−17971.18	516.25
	Change in *β*_rao_	Biotic	−23185.59	0
		Climate	−23173.58	12.01
		Full	−23167.89	17.70

At the hectad scale, mean minimum temperature had the largest effect size on change in *β*_sim_ [median = −0.0155, CI = (−0.0226, −0.0084)]. Variance in minimum temperature also had a strong, negative effect [median = −0.0106, CI = (−0.0154, 0.0058)]. Although variance in minimum temperature also affected changes in *β*_rao_ [median = −0.1391, CI = (−0.2688, −0.0095)], the covariates with the strongest effect sizes were both related to variation in growing degree days, that is, variance in growing degree days [median = −0.2292, CI = (−0.4032, −0.0553)] and lag autocorrelation in growing degree days [median = 0.2006, CI = (0.1086, 0.2925)]. The frequency of extreme events in minimum temperature and growing degree days influenced the degree of change in *β*_sim_, but extreme events had no effect on changes in *β*_rao_ as the credible intervals always spanned zero.

Changes in *β*_rao_ were not explained by any of our covariates at either of the larger two scales as the credible intervals of the distributions always spanned zero. Species richness explained some of the variation in changes in *β*_sim_ in models at all three spatial scales, but the mean binomial variance only appeared important in models at the two larger scales [30 km: median = −0.0033, CI = (−0.0042, −0.0024); 90 km: median = −0.0014, CI = (−0.0019, −0.0009)]. The covariate with the largest effect size for changes in *β*_sim_ at the 30 × 30 km scale was the relative number of extreme events in minimum temperature [median = −0.0074, CI = (−0.009, −0.0058)], while at the 90 × 90 km scale it was the long‐term trend in growing degree days [median = −0.0068, CI = (−0.0097, −0.0039)].

## 4 DISCUSSION

Taxonomic and functional homogenization of birds appears to be happening globally at a large scale, driven by human influences (Barnagaud et al., 2017); however, using a null model approach, we show that the occurrence of functional homogenization among British birds is not consistent given changes in taxonomic turnover at multiple spatial scales. The occurrence of functional homogenization varies with both spatial scale and measure of functional turnover (results for nearest functional neighbor and mean functional dissimilarity are presented in Supporting Information Appendix S2). At the largest spatial scale, in squares where changes in *β*_rao_ differ from the null expectation, the majority of squares show both taxonomic and functional homogenization, whereas at the smallest spatial scale, most squares show taxonomic and functional differentiation, although the overall number of squares where the null was rejected was considerably less at this scale.

Changes in taxonomic and functional turnover only appear to be correlated at the smallest spatial scale. Although this correlation is positive, nearly a quarter of hectads show taxonomic homogenization with either no associated change in *β*_rao_ or functional differentiation. Taxonomic homogenization was also more frequently accompanied by functional differentiation than functional homogenization at the 30 × 30 km scale. This suggests that despite areas becoming more compositionally similar, trait diversity is maintained. The impact of losing or gaining species on ecosystem functioning depends on the degree of trait overlap between species within a community (Baiser & Lockwood, 2011). Our results suggest a high level of trait overlap within a hectad, known as functional redundancy, as although species composition is becoming more similar, trait heterogeneity between squares is maintained, or, in some cases, even differentiates. This is consistent with Monnet et al. (2014) who found that temporal changes in taxonomic *β* diversity were not necessarily accompanied by corresponding changes in either functional or phylogenetic *β* diversity of avian communities in France. The same dataset, however, has revealed functional homogenization in response to urbanization (Devictor et al.,^2007^), although this study used a homogenization index of generalist and specialist species to measure functional homogenization, which may account for the contrasting results. This highlights the need to widen the focus from specialization when investigating functional homogenization and consider it in the context of temporal changes in the spatial dissimilarity of communities, as outlined by Olden et al. (2004).

Despite observations that changes in occurrences of British butterfly species between 1970–1982 and 1995–1999 can be deduced from spatial distribution patterns (Wilson, Thomas, Fox, Roy & Kunin, 2004), as well as theoretical and empirical evidence for the influence of biodiversity on biotic homogenization (Doxa, Paracchini, Pointereau, Devictor & Jiguet, 2012; Olden & Poff, 2003; Villéger et al., 2014), species richness and mean binomial variance were only included in the best performing model for changes in taxonomic turnover at the two larger spatial scales, showing negative relationships. Our hypothesis that biotic variables drive taxonomic homogenization, while climatic variables drive functional homogenization is therefore opposed at the smallest spatial scale. At larger spatial scales, however, our results support our predictions that squares where all occurring species are either locally rare or locally common showed more negative changes in *β*_sim_, that is, taxonomic homogenization. The variation in results with spatial scale may represent the scale at which dispersal becomes a limiting factor to a species’ distribution. Additionally, using a more recent bird atlas (i.e., Gillings, Balmer & Fuller, 2015) to study changes over a longer and more recent time period, such as that studied by Wilson et al. (2004), may yet reveal greater changes in turnover driven by measures of the spatial structure of distributions. The impact of these variables may also work at the species level, determining changes in species distributions, as shown in British and French butterflies where the signal was lost when combined into a community‐level study (Wilson et al., 2004).

Although no covariate had an opposing effect on changes in taxonomic and functional turnover, unlike Barnagaud et al. (2017), many covariates only appear important for changes in one type of turnover. For example, at the hectad scale, although both mean minimum temperature and mean drought show an association with changes in *β*_sim_, no mean measures showed any relationship with changes in *β*_rao_. Crucially, none of our covariates explained variation in change in *β*_rao_ at either of the larger spatial scales. At the smallest scale, however, the strongest climatic drivers differ between changes in *β*_sim_ and changes in *β*_Rao_; temperature is important for changes in taxonomic turnover, while growing degree days are important for changes in functional turnover, despite concurrent changes in community specialization of British birds with changes in both mean temperature and mean rainfall over a 13‐year period (Davey et al., 2012). Specifically, our results show that, at the hectad scale, mean minimum temperature drives the largest changes in *β*_sim_, while variance in growing degree days drives the largest changes in *β*_rao_. Overall, variation in growing degree days appears to promote functional homogenization as greater homogenization occurs in areas where there is increased variance and less year‐to‐year predictability. Growing degree days has been included in climate envelope models of avian distributions a number of times (Beale et al., 2014; Gregory et al., 2009; Huntley, Collingham, Willis & Green, 2008), as it represents the thermal energy available during the growing season and therefore is linked to resource availability (Huntley et al., 2008). Our results suggest that the variation in this energy at the hectad scale selects directly on species traits and, thus, spatial heterogeneity of functional diversity. Homogenization is occurring where resource availability is unstable. At larger spatial scales, temporal variation is also important to changes in *β*_sim_ with steeper long‐term trends (i.e., larger changes over the entire time period) and greater relative number of extreme events of multiple climatic components appearing to increase the degree of taxonomic homogenization. The observed relationships of changes in taxonomic and functional turnover with climatic variance, year‐to‐year predictability, long‐term trends, and extreme events measures at various scales show that more negative changes in turnover, that is, homogenization, occur in unstable environments, while a stable environment maintains spatial turnover and promotes more positive changes. This contrasts with the findings of Martin and Ferrer (2015), who showed that for Mediterranean birds, mammals, amphibians, and reptiles, temporally variable environments maintained higher spatial turnover; however, it supports the suggestion that environmental disturbances contribute to community homogenization through niche selection of disturbance‐tolerant species (Myers, Chase, Crandall & Jiménez, 2015). Environmental stability–spatial turnover relationships appear to be sparse within the literature, and the results found in the present paper open up a potential area of future research to further investigate this relationship.

The “difference in turnover” approach we employed for this study may help identify additional drivers of functional homogenization, such as land degradation. Results using mean community specialization have varied; for example, avian community specialization increased along a forest–agricultural gradient (Clavero & Brotons, 2010) but decreased along an urbanization gradient (Devictor et al., 2007). While the effects of changes in climate and land use have often been considered separately, their combined effects should also be considered when studying the impact of environmental change on ecological communities (Oliver & Morecroft, 2014). Building the framework used in this paper into investigations of the effects of multiple components of environmental change, along with their interactions, may further improve our predictions of areas susceptible to taxonomic and functional homogenization. Nonnative species may also contribute to functional homogenization, as they have been frequently implicated in taxonomic homogenization (Lockwood, Brooks & McKinney, 2000; McKinney & La Sorte, 2007; Rooney, Wiegmann, Rogers & Waller, 2004). We excluded nonnative and vagrant species from our analyses which focussed on changes in turnover of native and naturalized species. Despite this, there was evidence of both taxonomic and functional homogenization in a number of areas, indicating that mechanisms contributing to biotic homogenization are multifactorial: targeting invasive species alone will not prevent increasing community similarity of either species or traits.

Functional diversity is an important axis of biodiversity which dictates ecosystem functioning (Clark, Flynn, Butterfield & Reich, 2012; Díaz et al., 2007), community responses to environmental change (Ernst, Linsenmair & Rödel, 2006; Forrest, Thorp, Kremen & Williams, 2015; Meynard et al., 2011), and community stability and resilience (Mori, Furukawa & Sasaki, 2013). Using spatial (dis)similarity represents a recent approach to investigating functional homogenization and makes it more comparable with measures of taxonomic homogenization than previously used measures of mean community specialization. The changes in functional turnover independent of changes in taxonomic turnover evident in British avifauna reinforce the assertion that local colonizations and extinctions do not necessarily result in an associated change in trait composition (Baiser & Lockwood, 2011), and it is ill‐advised, therefore, to make any predictions about changes in ecosystem functioning based on the identification of taxonomic homogenization alone. The mechanisms driving taxonomic and functional homogenization are multifactorial, and actions taken to mitigate homogenization and its ecological consequences must account for this. Both functional diversity and species richness should be considered when planning habitat conservation to protect areas vulnerable to functional homogenization. Understanding the link between climate and homogenization will also help inform predictions of biodiversity responses to future climate projections. This study begins to build a comprehensive checklist of factors that increase the susceptibility of avian communities to homogenization at multiple spatial scales, which can be added to with further analyses on community composition, land cover, and invasive species potential.

## AUTHOR CONTRIBUTIONS

H.J.W. and J.J.L. conceived the idea for this manuscript. J.J.L. calculated the climatic variables. L.S. and D.H. provided the European Bird Trait Database. H.J.W. carried out the data analysis. H.J.W. led the writing with all authors contributing.

## DATA ACCESSIBILITY

Access to *The Atlas of Breeding Birds in Britain and Ireland* and *The New Atlas of Breeding Birds in Britain and Ireland: 1988–1991* for species occurrence data is available at https://data.nbn.org.uk/. Temperature and precipitation data are available for download from the UK MET office (metoffice.gov.uk/climatechange/science/monitoring/ukcp09). The European Bird Trait Database is in press at Global Ecology and Biogeography.
