# Supplementary material for: Does functional homogenization accompany taxonomic homogenization of British birds and how do biotic factors and climate affect these processes?
Source: Ecol Evol. 2018 Jun 27;8(15):7365–77. doi: 10.1002/ece3.4267 (PMC6106174; doi:10.1002/ece3.4267)
Supplement: Supplementary file 1 [file ECE3-8-7365-s001.docx]

**Supporting Information**

**Appendix S1.** Additional measures of functional turnover

Functional turnover was additionally calculated using two distance-based measures; nearest functional neighbour (NFN) and mean functional dissimilarity (Swenson et al., 2012; Sonnier et al., 2014). Gower distances were used to measure the functional distance between species in neighbouring communities. This distance measure can be used for both continuous and categorical traits, the latter of which were weighted according to the number of categories for that particular trait so that all traits contributed equally overall (Laliberté and Legendre, 2010). The first measure takes each species within one community (A) and finds the nearest functional neighbour within a neighbouring community (B) as well as the reverse (nearest functional neighbour in A of each species in community B). These values are averaged to find the mean nearest functional neighbour distance between neighbouring communities:

$$D_{nfn}= \frac{\sum_{i=1}^{nA} min \delta_{iB} + \sum_{j=1}^{nB} min\delta_{jA}}{2}$$

where $min \delta_{iB}$is the nearest functional neighbour of species *i* in community *A* in community *B* and $min\delta_{jkA}$is the nearest functional neighbour of species *j* in community *B* in community *A*. This metric is better able to detect subtle changes in trait composition between communities than other pairwise metrics (Swenson et al., 2012).

The mean functional dissimilarity measure calculates the mean pairwise functional distance of all species in community A to all species in community B:

$$D_{dis}= \frac{\sum_{i=1}^{nA} \bar{\delta_{iB}} + \sum_{j=1}^{nB} \bar{\delta_{jA}}}{2}$$

where $\bar{\delta_{iB}}$ is the mean pairwise functional distance between species *i* in community *A* and all the species in community *B*, and $\bar{\delta_{jA}}$is the mean pairwise functional distance between species *j* in community *B* in all of the species in community *A*. Swenson et al. (2012) suggest that this measure is better at detecting major trait turnover between communities than the nearest functional neighbour measure. In both measures of functional turnover, community A was assigned as the focal 10 km x 10 km square and community B as one of its neighbouring squares. Both calculations were calculated for the focal square and each of its neighbours (maximum 8) and the mean of each taken. We applied a moving-window algorithm so that each square within the dataset was included as the focal square in the calculations of turnover for both of the atlases.

**Appendix S2.** Results of occurrence of functional homogenisation using nearest functional neighbour and mean dissimilarity


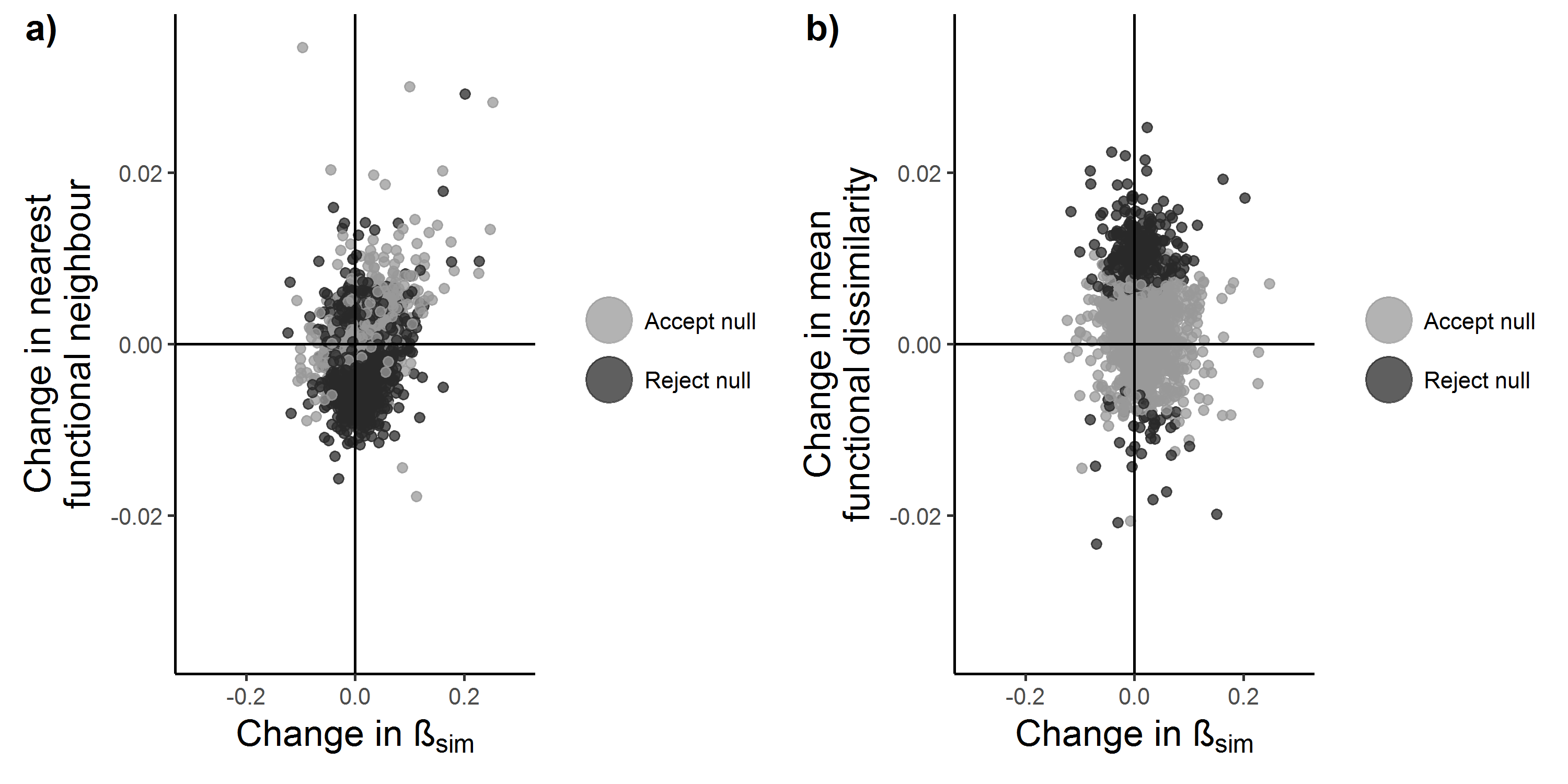


**Fig. S1.** Scatter plot of change in a) mean nearest functional neighbour and b) mean functional dissimilarity plotted against change in taxonomic turnover (β_sim_). Nearest functional neighbour and mean functional dissimilarity are both measures of functional turnover. Each point represents a 10 x 10 km square. Dark points indicate squares where the change in functional turnover is different from that expected from the null distribution of change in functional turnover generated from a null model of 999 randomisations of the species-by-trait matrix.

**Table S1.** Contingency table showing counts of hectads which showed larger than expected changes in nearest functional neighbour between two breeding bird atlases (null hypothesis was rejected). Hectads are split into those that show taxonomic homogenisation (decrease in β_sim_), taxonomic differentiation (increase in β_sim_), functional homogenisation (decrease in mean nearest functional neighbour) and functional differentiation (increase in mean nearest functional neighbour).

|  | Functional homogenisation | Functional differentiation |
| --- | --- | --- |
| Taxonomic homogenisation | 409 | 101 |
| Taxonomic differentiation | 707 | 176 |

**Table S2.** Contingency table showing counts of hectads which showed larger than expected changes in mean functional dissimilarity between two breeding bird atlases (null hypothesis was rejected). Hectads are split into those that show taxonomic homogenisation (decrease in β_sim_), taxonomic differentiation (increase in β_sim_), functional homogenisation (decrease in mean functional dissimilarity) and functional differentiation (increase in mean functional dissimilarity).

|  | Functional homogenisation | Functional differentiation |
| --- | --- | --- |
| Taxonomic homogenisation | 13 | 179 |
| Taxonomic differentiation | 27 | 256 |

**
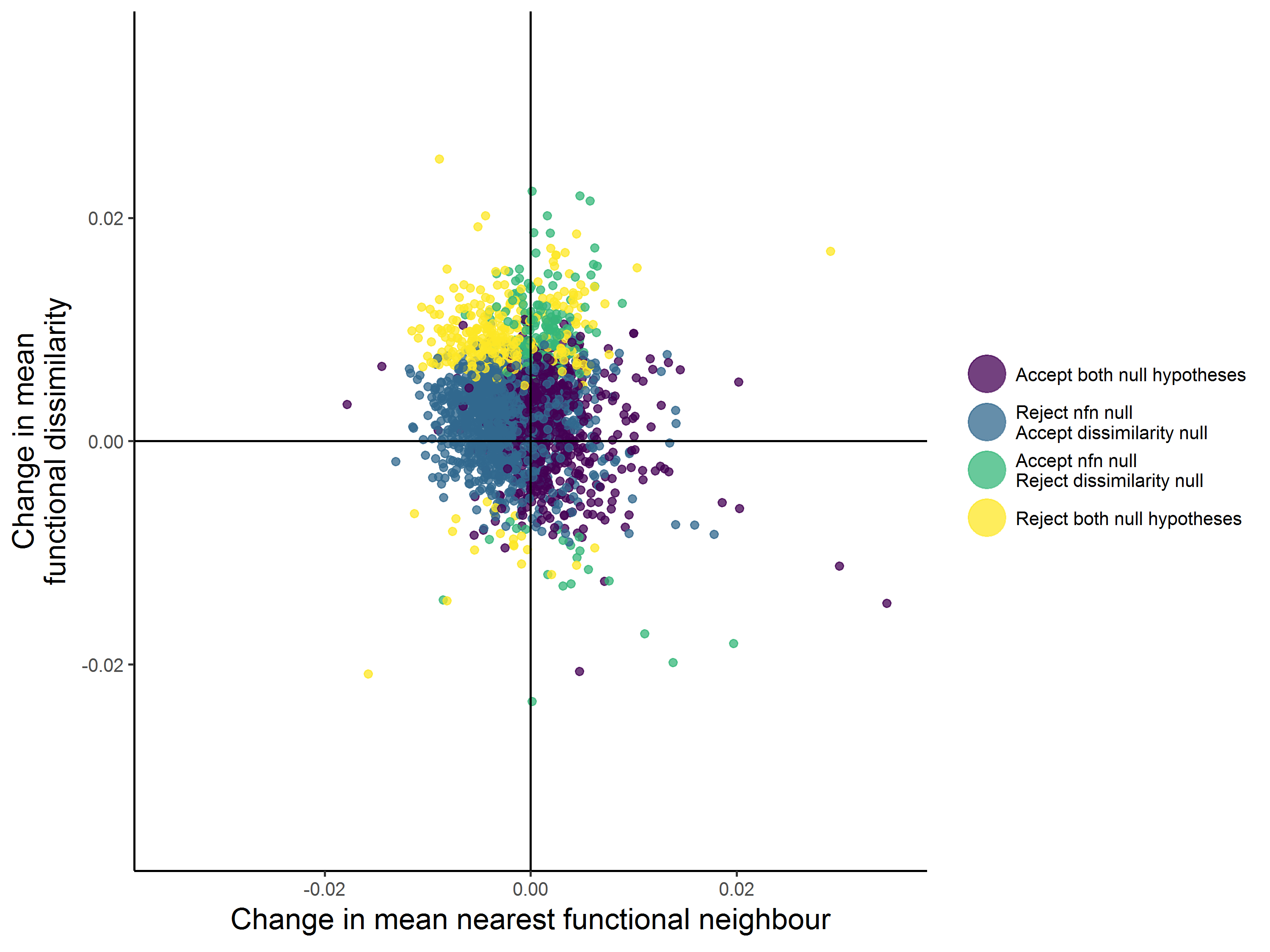
**

**Fig. S2.** Scatter plot of change in mean functional dissimilarity, plotted against change in mean nearest functional neighbour. Each point represents a 10 x 10 km square, coloured according to whether the change in each measure of functional turnover was different than expected from random given a null model of 999 randomisations of the species-by-trait matrix. nfn =nearest functional neighbour, dissimilarity = mean functional dissimilarity.
